# Retroviral expression of human arginine decarboxylase reduces oxidative stress injury in mouse cortical astrocytes

**DOI:** 10.1186/1471-2202-15-99

**Published:** 2014-08-26

**Authors:** Samin Hong, Mi Ran Son, Kyungeun Yun, Won Taek Lee, Kyung Ah Park, Jong Eun Lee

**Affiliations:** Institute of Vision Research, Department of Ophthalmology, Yonsei University College of Medicine, Seoul, Republic of Korea; Brain Korea 21 Project for Medical Science, and Brain Research Institute, Department of Anatomy, Yonsei University College of Medicine, 50 Yonsei-ro, Seodaemun-gu, Seoul, 120-752 Republic of Korea

**Keywords:** Agmatine, Arginine decarboxylase, Astrocyte, Neuroprotection, Oxidative stress

## Abstract

**Background:**

In physiologic and pathologic conditions of the central nervous system (CNS), astrocytes are a double-edged sword. They not only support neuronal homeostasis but also contribute to increases in neuronal demise. A large body of experimental evidence has shown that impaired astrocytes play crucial roles in the pathologic process of cerebral ischemia; therefore, astrocytes may represent a breakthrough target for neuroprotective therapeutic strategies. Agmatine, an endogenous polyamine catalyzed from L-arginine by arginine decarboxylase (ADC), is a neuromodulator and it protects neurons/glia against various injuries.

**Results:**

In this investigation, agmatine-producing mouse cortical astrocytes were developed through transduction of the human ADC gene. Cells were exposed to oxygen-glucose deprivation (OGD) and restored to a normoxic glucose-supplied condition. Intracellular levels of agmatine were measured by high performance liquid chromatography. Cell viability was evaluated by Hoechest/propidium iodide nuclear staining and lactate dehydrogenase assay. Expression of inducible nitric oxide synthase (iNOS) and matrix metalloproteinase s (MMPs) were assessed by a reverse transcription polymerase chain reaction, Western immunoblots, and immunofluorescence. We confirmed that ADC gene-expressed astrocytes produce a great amount of agmatine. These cells were highly resistant to not only OGD but also restoration, which mimicked ischemia-reperfusion injury *in vivo*. The neuroprotective effects of ADC seemed to be related to its ability to attenuate expression of iNOS and MMPs.

**Conclusion:**

Our findings imply that astrocytes can be reinforced against oxidative stress by endogenous agmatine production through ADC gene transduction. The results of this study provide new insights that may lead to novel therapeutic approaches to reduce cerebral ischemic injuries.

**Electronic supplementary material:**

The online version of this article (doi:10.1186/1471-2202-15-99) contains supplementary material, which is available to authorized users.

## Background

Astrocytes comprise the structural architecture of the brain and support neurons to maintain homeostasis [1, 2]. Physiologically, they stabilize the extracellular environment of neurons: they control ion/water distribution, regulate neurotransmitter recycling, release growth factors, and scavenge reactive oxygen species (ROS). Under pathologic conditions, when noxious stress exceeds limitations, astrocytes work in the opposite direction [1, 2]. Water accumulation induces brain edema, failure of neurotransmitter control, which causes glutamate excitotoxicity; and releases ROS/neurotoxic factors that contribute to further neuronal damage. In cerebral injuries, astrocytes are the main culprit of neurodegeneration and scar formation [2, 3]. Meanwhile, they also contribute to neurite outgrowth and neurogenesis [4]. Astrocytes have recently garnered attention as a breakthrough target in development of new therapeutic strategies for patients suffering from neuronal damage [2].

Agmatine, an endogenous polyamine derived from L-arginine by arginine decarboxylase (ADC), is naturally found in the mammalian central nervous system (CNS) and acts as a multifunctional neuromodulator [5–7]. It is packed into synaptic vesicles and released from synaptosomes by neuronal depolarization [8, 9]. Agmatine can stimulate α_2_-adrenergic and imidazoline receptors [10, 11], block the N-methyl-D-aspartic acid (NMDA) receptor and voltage-gated calcium channel [12, 13], and inhibit inducible/neuronal nitric oxide synthases (iNOS/nNOS) [14, 15]. Through these kinds of intracellular signaling, agmatine shows various antineurotoxic, anticonvulsant, antipsychotic, antidepressant, anxiolytic, and antinociceptive neuroprotective effects [7, 16]. Regarding ischemic insults, exogenous agmatine significantly reduces the infarct size and brain edema caused by ischemia-reperfusion injury in rodents [17–20]. Agmatine also rescues glial cells, as well as cortical neurons, from oxidative stress *in vitro* and *in vivo* [17, 21, 22].

In this study, a recombinant retroviral vector containing the human ADC (hADC) gene was introduced into mouse cortical astrocytes to maximize the astrocyte-protective effect of agmatine. Cells overexpressing hADC show increased production of agmatine and greater resistance against oxidative stress [23–25]. Here, we transduced primary cultured cortical astrocytes with hADC-expressing retroviral vector and assessed whether it induces synthesis of endogenous agmatine and attenuates oxidative stress injuries.

## Methods

### Cell culture

Primary cortical astrocyte cultures were prepared from postnatal 1–3 days old Crl:CD1 mouse pups (Samtako, Osan, Korea) [21, 26]. Our protocol was approved by the Institutional Animal Care and Use Committee of Yonsei University College of Medicine and all animals were treated in accordance with their guidelines. Animals were etherized and decapitated on ice-cold ethanol. Cortical hemispheres were dissected free of the skull and meninges, and then treated with 0.09% trypsin for 20 min at 37°C. The cells were mechanically dissociated into single cells and collected as a suspension. The cells were grown in minimum essential medium (MEM; Life Technologies, Grand Island, NY) containing 10% fetal bovine serum (FBS) and 10% equine serum (Thermo Fisher Scientific, Waltham, MA). The cultures were incubated at 37°C in a 5% CO_2_ incubator and the culture media was replaced every 3 days. One week after the first seeding, the other cells including microglia were removed by vigorous shaking. The highly-purified astrocytes (passage 3), at least 3 weeks after the first seeding, were used for experiments at about 80% confluence (Additional file 1).

### Construction and infection of recombinant retroviral vector containing the hADC gene

**Figure 1:**
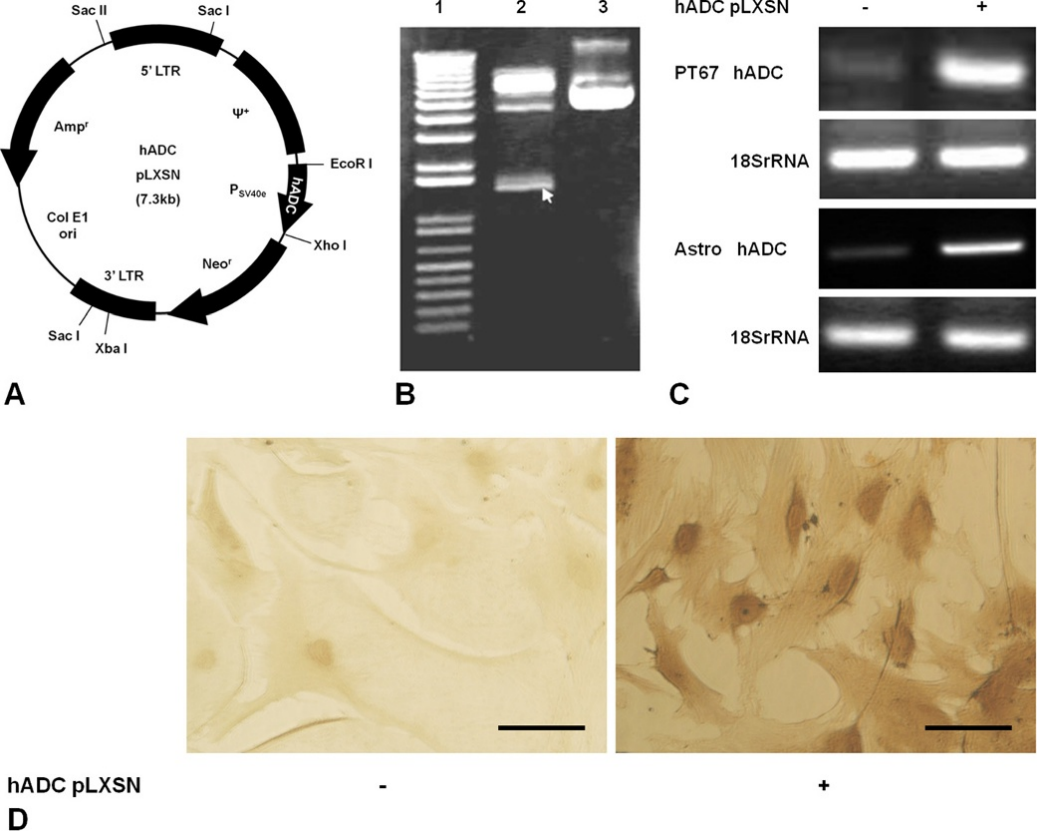
**Construction and infection of recombinant retroviral vector containing the human arginine decarboxylase (hADC) gene. (A)** hADC pLXSN vector map: hADC pLXSN includes the Col E1 origin of replication and *E.coli* Amp^r^ gene for propagation and antibiotic selection. The 5' viral LTR in this vector contains promoter/enhancer sequences that control expression of the gene of interest in the multiple cloning site. The SV40 early promoter (PSV40_e_) controls expression of the neomycin resistance gene (Neo^r^), which allows antibiotic selection in eukaryotic cells. **(B)** Genetic confirmation by restriction analysis: Lane 1, size marker; Lane 2, restriction enzyme digestion using EcoRI and XhoI; Lane 3, control hADC pLXSN. **(C)** RNA levels of hADC were verified by RT-PCR in PT67 cells and mouse cortical astrocytes (Astro) after hADC pLXSN infection. **(D)** Expression of hADC was validated by immunocytochemistry in hADC pLXSN-infected astrocytes. Scale bar = 50 μm.

A recombinant retroviral vector containing the hADC gene was constructed as described previously (Figure 1) [23–25]. Briefly, the full-length cDNA of hADC (GenBank accession no. AY325129) was amplified by a polymerase chain reaction (PCR) and ligated to the retroviral expression vector pLXSN (Clontech Laboratories, Mountain View, CA) that also contained a neomycin resistance gene. They were then amplified in *E.coli* DH5a and identified by restriction analysis. The hADC-expressing pLXSN vector was transfected into the retroviral packaging cell line PT67 using Lipofectamine 2000 (Sigma-Aldrich, St. Louis, MO). A stable clone selection was carried out by adding G418 (200 μg/mL; Sigma-Aldrich) to the culture medium of Dulbecco’s modified Eagle’s medium (DMEM, Life Technologies) supplemented with 10% FBS. High-titer clones were selected, and virus-containing supernatant was filtered through a 0.45-mm polysulfonic filter. Primary cultured mouse cortical astrocytes were infected with hADC pLXSN. The cells were incubated with the virus-containing media for 24 hrs and then maintained in normal culture medium for a week before being used for the experiments.

### Oxygen-glucose deprivation and restoration

For oxygen-glucose deprivation (OGD), cultures were transferred to a closed anaerobic chamber (Forma Scientific Co., Seoul, Korea) in which the oxygen level (less than 0.1% O_2_) and temperature (37°C) were controlled automatically [21, 27]. After being washed three times with deoxygenated, glucose-free balanced salt solution (BSS_0_) at pH 7.4, cells were kept in the anaerobic chamber for 4 hours. Cultures were then restored with glucose at a concentration of 5.5 mM (BSS_5.5_) and recovered to normoxic conditions (37°C, 5% CO_2_) for up to 10 hours.

### High performance liquid chromatography analysis

The concentration of agmatine was measured by high performance liquid chromatography (HPLC) [23, 24]. Harvested cells were homogenized in phosphate buffer containing 10% trichloroacetic acid and centrifuged at 12,000 rpm for 10 min. The collected supernatant was derivatized with o-phthalaldehyde and loaded onto the HPLC column (5 mm) connected with a fluorescence detector. Recovery of agmatine was calculated from an added external standard. The content of L-Arginine and putrescine was also measured using same method.

### Hoechst and propidium iodide nuclear staining

Cell death was characterized by double nuclear staining with Hoechst 33258 and propidium iodide (PI) [23, 24]. Cells were stained with Hoechst 33258 dye (5 μg/mL; Sigma-Aldrich) for 30 min at 37°C and consecutively stained with PI (5 μg/mL; Sigma-Aldrich) just before observing it with a fluorescence microscope. Living cells showed Hoechst-positive blue fluorescence and nonviable cells had PI-positive red fluorescence.

### Lactate dehydrogenase activity

Cell viability was quantified by measuring the amounts of lactate dehydrogenase (LDH) released from injured cells into the medium [27–30]. LDH released (% cytotoxicity) was calculated by dividing the value at the experimental time point by the maximum value × 100. Maximum LDH release values were obtained after freezing each culture at −70°C overnight and then rapidly thawing them, thus inducing nearly complete cell damage.

### Reverse transcription polymerase chain reaction

Gene expression was assessed by reverse transcription PCR (RT-PCR) [23, 24]. Total RNA was extracted from cells with Trizol (Life Technologies) and quantified by measuring the absorbance at 260 nm. cDNA synthesis from mRNA and normalization of the samples were carried out by RT-PCR. PCR amplification of iNOS was performed at 94°C for 30 s, 57.5°C for 30 s, and 72°C for 30 s for 30 cycles. Amplification of MMP-2 was performed at 94°C for 30 s, 53°C for 30 s, and 72°C for 30 s for 35 cycles. Amplification for MMP-9 was performed at 94°C for 30 s, 59°C for 30 s, and 72°C for 30 s for 30 cycles. Specific primer sequences used are as follows: iNOS, sense 5'-TTT GAT GTG CTG CCT CTG GT-3' and antisense 5'-CAT TCT GCT TCT GGA AAC TAA GG-3'; MMP-2, sense 5'-GAG TTG GCA GTG CAA TAC CT-3' and antisense, 5'-GCC GTC CTT CTC AAA GTT GT-3'; MMP-9, sense 5'-TTA CCA GCG CCA GCC GAC TTT TG-3' and antisense 5'-CGT CGT CGT CGA AAT GGG CAT C-3'; Glyceraldehyde 3-phosphate dehydrogenase (GAPDH), sense 5'-ATG TCG TGG AGT CTA CTG GT-3' and antisense 5'-TGG CAT GGA CTG TGG TG-3'. PCR products were separated by electrophoresis in a 1.5% agarose gel stained with ethidium bromide.

### Western immunoblots

Protein levels were determined by Western immunoblots [23, 24, 31]. Protein was extracted from whole cell lysates, and their concentration was calculated with the BCA protein assay (Thermo Fisher Scientific). Equal amounts of protein (40 μg) from each sample were resolved on a 10% sodium dodecyl sulfate-polyacrylamide gel. The separated proteins were then electrotransferred onto Immobilon-NC membranes (Millipore, Billerica, MA) and incubated overnight with primary antibodies against iNOS (1:1000 dilution), MMP-2 (1:2000 dilution), MMP-9 (1:2000 dilution), or β-actin (1:2000 dilution). The primary antibody against iNOS was purchased from Millipore (Billerica, MA) and the others were obtained from Santa Cruz Biotechnology (Dallas, TX). Immunoreactive bands were detected with horseradish peroxidase-conjugated secondary antibody and visualized using an enhanced chemiluminescent protein detection kit (Thermo Fisher Scientific).

### Immunofluorescence staining

Protein expression was also confirmed by immunofluorescence [23, 31, 32]. Cells were fixed with 90% ethanol for 30 min on ice, and then treated with 1.6% hydrogen peroxide and 0.025% triton. They were reacted with specific antibodies (1:500 dilution) for iNOS (Millipore), MMP-2 (Santa Cruz Biotechnology), MMP-9 (Santa Cruz Biotechnology), and glial fibrillary acidic protein (GFAP; Abcam, Cambridge, MA). Subsequently, the cells were exposed to the corresponding fluorescent secondary antibodies (Life Technologies) and their nuclei were counterstained with 4’,6-diamidino-2-phenylindole (DAPI; Sigma-Aldrich). Finally, four random fields were imaged under a fluorescence microscope. Immunoreactive cells with fluorescence were manually counted at 200× magnification.

### Statistical analysis

Data are presented at the mean of ± SD of at least three different experiments performed from separate cell preparations, with at least triplicate determination performed for each experiment. Kruskal-Wallis test and Mann–Whitney test were used in order to examine statistical significance (MedCalc Statistical Software version 12.7.8.; MedCalc Software bvba, Ostend, Belgium). P-values less than 0.05 were considered statistically significant.

## Results

### Transduction of mouse cortical astrocytes with recombinant retroviral vector containing the human arginine decarboxylase (hADC) gene

The vector map of hADC pLXSN is shown in Figure 1A. It includes the Col E1 origin of replication and *E.coli* Amp^r^ gene for propagation and antibiotic selection. The 5' viral LTR contains promoter/enhancer sequences that control expression of the gene of interest in the multiple cloning site. The SV40 early promoter (PSV40_e_) controls expression of the neomycin resistance gene (Neo^r^), which allows for antibiotic selection in eukaryotic cells. PSV40_e_ was genetically confirmed by restriction analysis (Figure 1B). In astrocytes, as well as PT67 cells, infection of the hADC pLXSN vector effectively induced hADC transcription (Figure 1C). Expression of hADC was validated by immunocytochemistry in hADC pLXSN-infected astrocytes (Figure 1D).

### Endogenous agmatine production from human arginine decarboxylase (hADC)-overexpressed astrocytes

**Figure 2:**
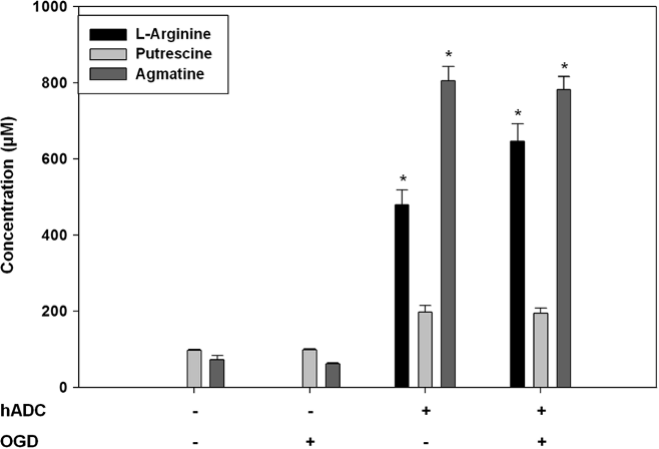
**Endogenous agmatine measurement using high performance liquid chromatography (HPLC).** The level of amines related to the agmatine pathway was determined in mouse cortical astrocytes infected with retrovirus containing the human arginine decarboxylase (hADC) gene. Cells underwent oxygen-glucose deprivation (OGD) for 4 hrs. Data are expressed as a mean ± SD of three different experiments performed from separate cell preparation, with triplicate determinations performed in each experiment. Asterisks indicate a p < 0.001 when the samples were compared to no treatment control.

Intracellular levels of agmatine were measured by HPLC (Figure 2). Endogenous agmatine concentration was markedly greater for astrocytes overexpressing hADC compared to that of control astrocytes (11.28-fold, p < 0.001). The amount of L-arginine and putrescine (precursor and metabolite of agmatine, respectively) showed similar tendency to that of agmatine. Meanwhile, OGD itself did not have a significant influence on the level of these amines.

### Astrocyte-protective effect of human arginine decarboxylase (hADC) transduction against oxygen-glucose deprivation (OGD)

**Figure 3:**
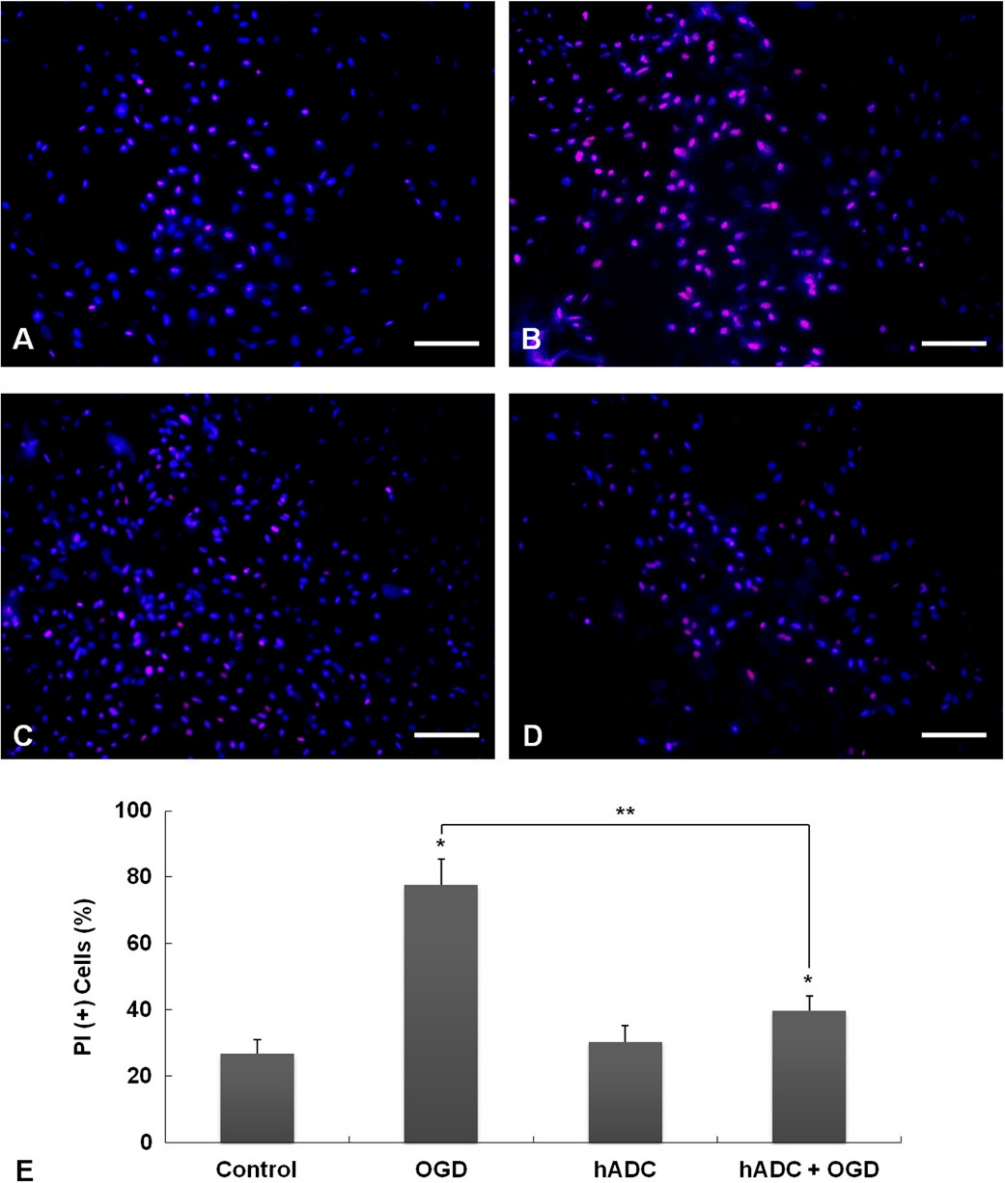
**Astrocyte-protective effect of human arginine decarboxylase (hADC) transduction against oxygen-glucose deprivation (OGD).** Cytotoxicity was assessed by Hoechst 33258 and propidium iodide (PI) nuclear staining; **(A)** No treatment control astrocytes, **(B)** astrocytes after 4 hrs OGD, **(C)** hADC-overexpressing astrocytes (hADC-astrocytes), **(D)** hADC-astrocytes after 4 hrs OGD. Scale bars = 100 μm. **(E)** Proportion of PI-labeled damaged cells with red fluorescence. Asterisks indicate a p < 0.05 when the samples were compared to no treatment control. Double asterisk indicate a p < 0.05 that that the two samples were significantly different which was confirmed by a post-hoc analysis.

OGD damaged naive astrocytes and the majority of cells had PI-labeled nuclei (Figure 3B). However, astrocytes overexpressing hADC (hADC-astrocytes) were much less damaged by OGD, and the proportion of PI-positive cells were considerably lower (Figure 3D). hADC transduction itself did not have any significant effects on cell viability (Figure 3C), as determined by Hoechst 33258 and PI double nuclear staining.

### Neuroprotective effect of human arginine decarboxylase (hADC) transduction on oxygen-glucose deprivation (OGD) and restoration injury

**Figure 4:**
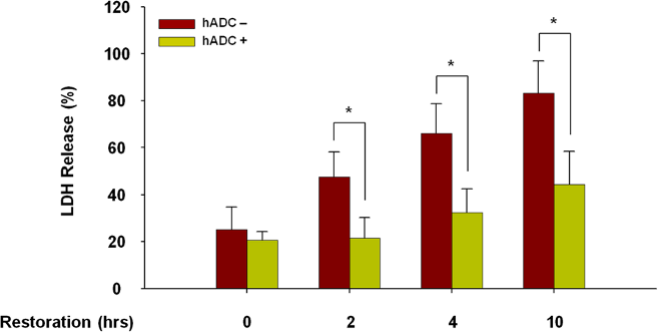
**Neuroprotective effect of human arginine decarboxylase (hADC) transduction against oxygen-glucose deprivation (OGD) and restoration injury.** Astrocytes restored to normoxic glucose-supplied conditions after 4 hrs OGD. Cytotoxicity was assessed by the lactate dehydrogenase (LDH) assay. Data are expressed as mean of ± SD of the three different experiments performed from separate cell preparations, with triplicate determinations performed in each experiment. Asterisks indicate a p < 0.001.

To mimic the ischemia-reperfusion injury *in vivo*, cells were restored to normoxic glucose-supplied conditions after 4 hrs of OGD (Figure 4). Cytotoxicity was measured by LDH leakage from the injured cells. Cytotoxicity gradually increased in a time-dependent manner for up to 10 hrs during restoration. Even in hADC-astrocytes, the restoration from OGD caused cytotoxicity in a time-dependent-manner; the extent of damage was much less than that in control astrocytes.

### Suppression of inducible nitric oxide synthase (iNOS) and matrix metalloproteinases (MMPs) in human arginine decarboxylase (hADC)-overexpressing astrocytes

**Figure 5:**
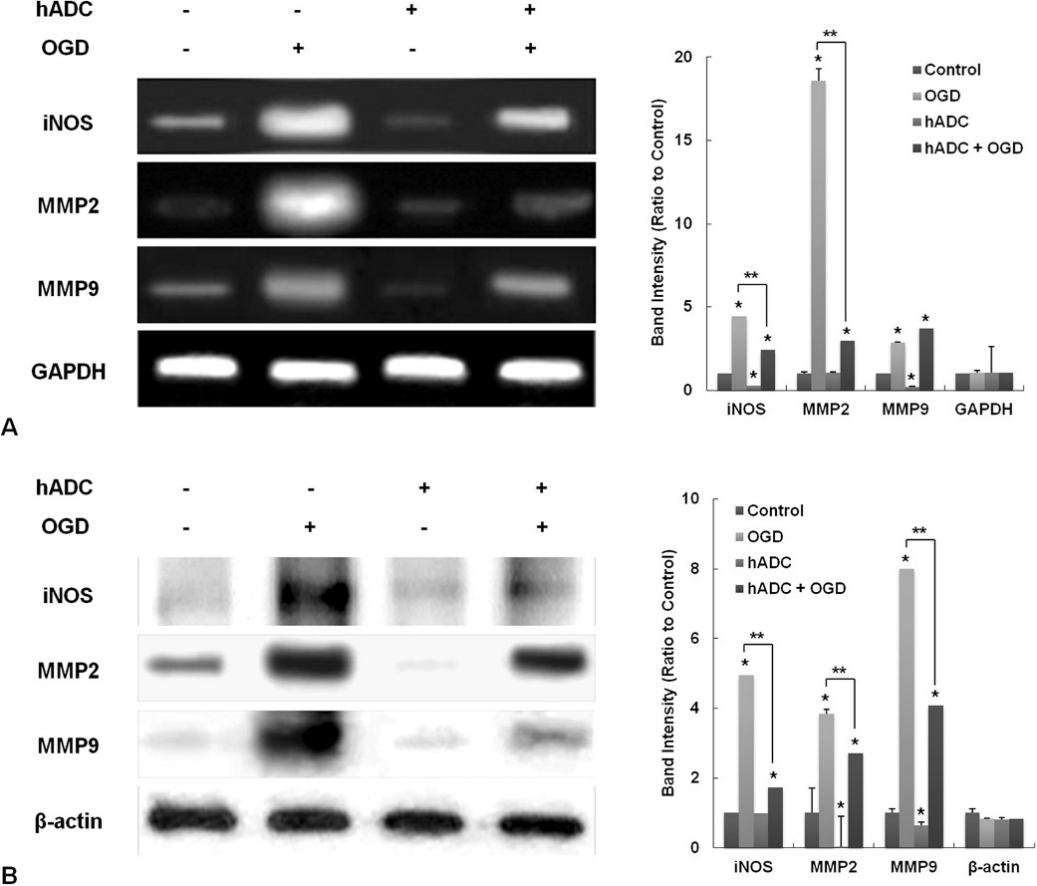
**Inducible nitric oxide synthase (iNOS) and matrix metalloproteinases (MMPs) expression in human arginine decarboxylase (hADC)-introduced astrocytes after oxygen-glucose deprivation (OGD).** The mRNA levels were determined using reverse transcription polymerase chain reaction (RT-PCR) **(A)** and protein expression was evaluated using Western immunoblots **(B)**. Glyceraldehyde 3-phosphate dehydrogenase (GAPDH) and β-actin were used as internal controls, respectively. Asterisks indicate a p < 0.05 when the samples were compared to no treatment control. Double asterisk indicate a p < 0.05 that two samples were significantly different which was confirmed by a post-hoc analysis.

Expression of iNOS and MMP-2/9 was assessed by RT-PCR (Figure 5A) and Western immunoblots (Figure 5B). OGD significantly increased levels of iNOS and MMP-2/9; however, hADC transduction attenuated the OGD effects. Introduction of the hADC gene itself did not change glyceraldehyde 3-phosphate dehydrogenase (GAPDH) and β-actin levels, which served as internal controls for RT-PCR and Western immunoblots, respectively.

### Inhibited expression of inducible nitric oxide synthase (iNOS) and matrix metalloproteinases (MMPs) in human arginine decarboxylase (hADC)-transduced astrocytes

**Figure 6:**
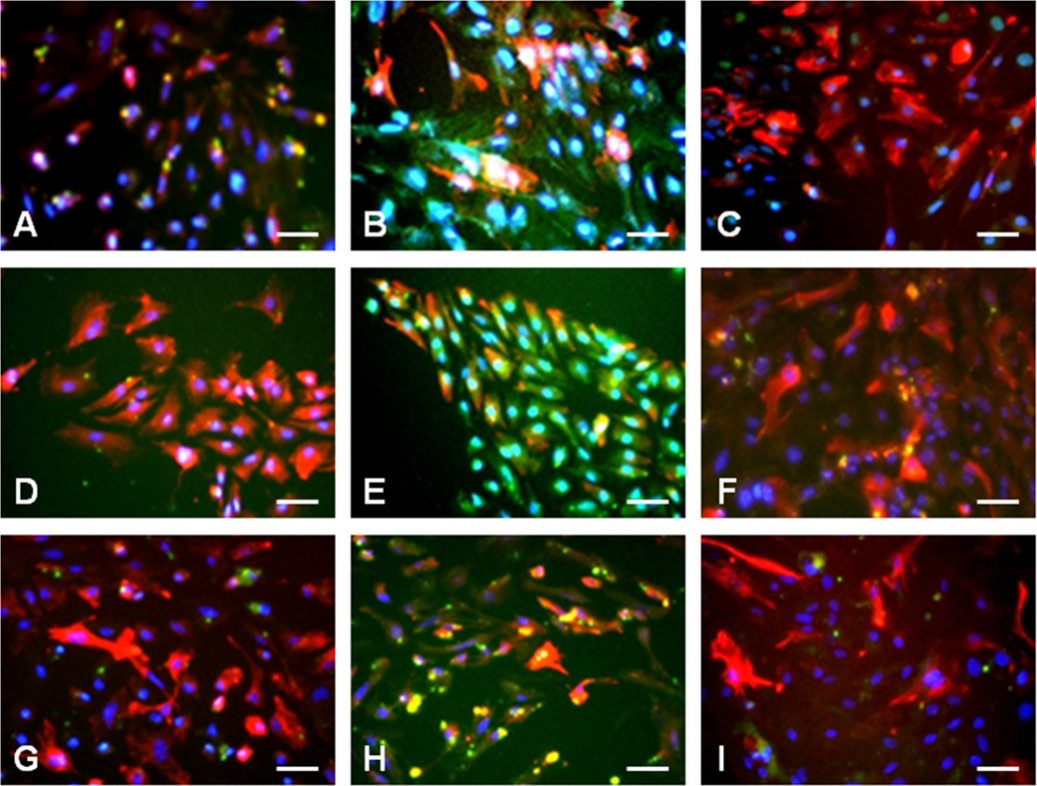
**Immunofluorescence staining of human arginine decarboxylase (hADC)-introduced astrocytes after oxygen-glucose deprivation (OGD).** **(A, D, G)** No treatment control astrocytes; **(B, E, H)** astrocytes after 4 hrs OGD; **(C, F, I)** hADC-astrocytes after 4 hrs OGD. Cells reacted to inducible nitric oxide synthase (iNOS) **(A-C)**, matrix metalloproteinase (MMP) 2 **(D-F)**, and MMP 9 **(G-I)** showed green fluorescence. Cells were counter-stained with astrocyte marker glial fibrillary acidic protein (GFAP; red fluorescence) and nuclear marker 4',6-diamidino-2-phenylindole (DAPI; blue fluorescence). Scale bar = 50 μm.

Naive astrocytes seldom expressed iNOS and MMP-2/9 (Figure 6A,D,G). After 4 hrs of OGD, control astrocytes exhibited expression of iNOS and MMP-2/9 (Figure 6B, E, H); however, hADC-astrocytes did not show immunoreactivity to either iNOS or MMP-2/9 (Figure 6C, F, I).

## Discussion

Since Reis and colleagues first discovered agmatine and ADC activity in the mammalian brain [5], agmatine has been widely studied. It is known to have various biological actions through multiple molecular targets, exhibiting affinity not only to α_2_-adrenoceptors and I_1_- and I_2_-imidazoline receptors but also to β-adrenoceptors and 5-HT_3_ serotonin, nicotinic cholinergic NMDA, D_2_-dopamine, κ-opioid, and adenosine A_1_ receptors [10–12]. Agmatine also inhibits iNOS/nNOS and monoamine oxidase [10, 14, 15]. As well, agmatine reduces heart rate and blood pressure [10], stimulates insulin and β-endorphin release, leading to increased cellular glucose uptake [33, 34], and accelerates glomerular ultrafiltration [35]. Agmatine protects systemic organs, especially against oxidative stress. Additionally, it shows cardioprotective and renoprotective effects on ischemic insult [36, 37], and it directly protects mitochondria from ROS [38, 39]. In the CNS, agmatine shows neuroprotective effects on various neuronal injuries: reduces the infarct size and edema after cerebral ischemia [17, 18, 40], attenuates brain damage and reactive gliosis caused by trauma [41, 42], and also decreases traumatic/ischemic spinal cord injuries [43, 44]. Agmatine also decreases neuropathic pain and convulsive events [45, 46], attenuates opioid dependence and alcohol withdrawal anxiety [47, 48], and it improves motor and cognitive functions in Parkinson's and Alzheimer's disease, respectively [49, 50]. Even in the eyes, agmatine lowers intraocular pressure and protects retinal ganglion cells [51, 52]. Taken together, agmatine seems to be a miracle cure rather than a specific drug.

In routine clinical settings, exogenous drug administration may be the most straightforward therapeutic strategy. However, exogenous drug administration is not always the best approach for real patients. Taken orally, agmatine is readily distributed throughout the body [53, 54] and can even cross the blood–brain barrier [55, 56]. Moreover, its reported half-life in the body is about 2 hrs [57]. The various functions of agmatine have pros and cons. In addition to neuronal effects, agmatine also has cardiovascular, endocrinal, renal, gastric, and growth effects [10]. Therefore, taken orally, agmatine may cause unwanted systemic responses. Thus, it is important to focus on the action of the drug at the site of injury and extend its duration of action. In this regard, in the ophthalmologic field, a topical agmatine ophthalmic solution has been formulated to preserve the optic nerves against glaucomatous damage [51]. Agmatine eye drops effectively lower intraocular pressure and protect retinal ganglion cells from chronic ocular pressure injuries. Along the same lines, investigators have attempted to develop a distinctive method for increasing endogenous agmatine production via a recombinant retroviral vector system containing the hADC gene. The hADC gene can be effectively delivered into mouse fibroblast NIH3T3 cells [23], primary mouse cortical neural stem cells [24], and human bone marrow mesenchymal stem cells [25]. Different types of transformed cells synthesize agmatine when they are exposed to hydrogen peroxide, resulting in resistance against oxidative stress. Irrespective of the cell type in an oxidative stressed environment, intracellular agmatine concentration is boosted by about 2 to 5-fold in hADC-overexpressing cells compared to no treatment control cells, as determined by HPLC [23–25]. While in normal culture conditions, agmatine synthesis is significantly increased in cortical neural stem cells [24] but not fibroblasts or mesenchymal stem cells [23, 25]. It is not yet clear why the activity of an introduced hADC gene differs according to cell type, especially in non-stressful conditions; nonetheless, it is assumed that agmatine controls the expression of the introduced hADC gene. If normal cells use little agmatine, the transformed cells are presumed not to utilize the introduced ADC without harmful stimuli. However, if normal cells actively use agmatine, the transformed cells are presumed to utilize the introduced ADC. When cells are attacked, all the cell types seem to use the introduced ADC.

In this investigation, we focused on cortical astrocytes, which have recently came to the forefront due to their suspected roles in many CNS injuries, including cerebral ischemia [2, 3]. Our results showed that intracellular levels of agmatine, as well as its precursor arginine and byproduct putrescine, are definitively increased (about 11-fold compared to the control, p < 0.001) in normal and oxidative stressed conditions upon hADC gene transduction, as measured by HPLC. Simultaneously, cell viability was determined by Hoechst /PI double nuclear staining and the LDH assay. hADC-overexpressing cells remained viable and healthy under oxidative stress conditions induced by OGD for 4 hrs. This astrocyte-rescuing property was gradually potentiated as restoration time proceeded for up to 10 hours. In addition, the neuroprotective effect of endogenous agmatine seemed to be related to iNOS intracellular signaling and the activity of MMPs, as assessed by RT-PCR, Western immunoblots, and immunofluorescence. Our present and previous findings are similar to those of other groups in regards to iNOS [15, 58–60] and MMPs [61, 62], respectively. As MMPs are upregulated by ischemic insult and degrade the basement membrane of brain microvessels [63], their suppression via ADC gene transduction may reduce ischemic injuries. Our retroviral system to deliver the hADC gene to target cells cannot be directly applied to non-dividing cells. Accordingly, another vector system using the Lentivirus is currently under development. It may be directly applied to non-dividing CNS neurons, specifically through *in vivo* transduction.

## Conclusion

In summary, our findings imply that endogenous agmatine production through ADC gene transduction can protect astrocytes against oxidative stress. The results of this study provide new insights that may lead to novel therapeutic approaches to reduce oxidative stress-related diseases including cerebral ischemia.

## Electronic supplementary material

Additional file 1: **Immunofluorescence staining of primary cultured astrocytes.** Cells reacted to anti-glial fibrillary acidic protein (GFAP) (A, red fluorescence), and anti-CD11b (B, green fluorescence) antibodies. Nuclei were counterstained with Hoechst 33258 (C, blue fluorescence). Panel D is a merged image. Scale bar = 200 μm. (TIFF 559 KB)

## Abbreviations

ADC: Arginine decarboxylase
ANOVA: One-way analysis of variance
BSS: Balanced salt solution
CNS: Central nervous system
DAPI: 4’,6-diamidino-2-phenylindole
DMEM: Dulbecco’s modified Eagle’s medium
FBS: Fetal bovine serum
GAPDH: Glyceraldehyde 3-phosphate dehydrogenase
GFAP: Glial fibrillary acidic protein
hADC: Human arginine decarboxylase
HPLC: High performance liquid chromatography
iNOS: Inducible nitric oxide synthase
LDH: Lactate dehydrogenase
MEM: Minimum essential medium
MMP: Matrix metalloproteinase
NMDA: N-methyl-D-aspartic acid
nNOS: Neuronal nitric oxide synthase
OGD: Oxygen-glucose deprivation
PCR: Polymerase chain reaction
PI: Propidium iodide
ROS: Reactive oxygen species
RT-PCR: Reverse transcription polymerase chain reaction.
