# Supplementary figures and images for: Retroviral expression of human arginine decarboxylase reduces oxidative stress injury in mouse cortical astrocytes
Source: BMC Neurosci. 2014 Aug 26;15:99. doi: 10.1186/1471-2202-15-99 (PMC4150973; doi:10.1186/1471-2202-15-99)

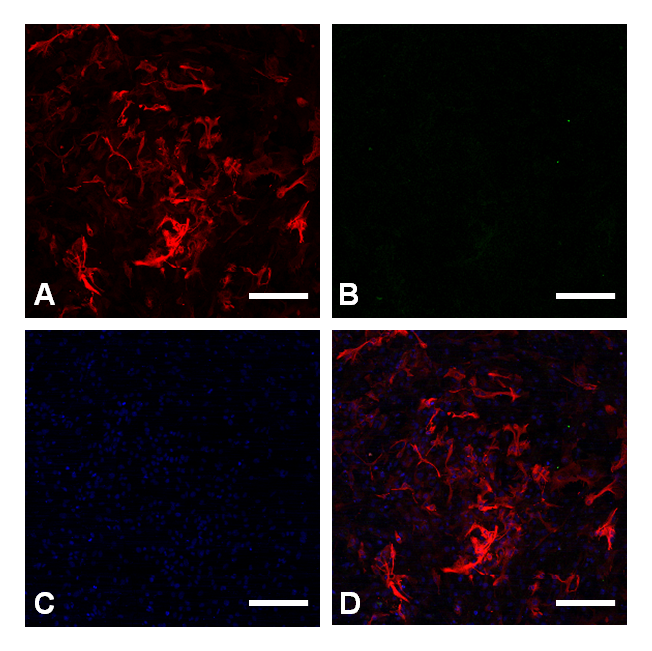

Supplement: Supplementary file 1 — Additional file 1: Immunofluorescence staining of primary cultured astrocytes. Cells reacted to anti-glial fibrillary acidic protein (GFAP) (A, red fluorescence), and anti-CD11b (B, green fluorescence) antibodies. Nuclei were counterstained with Hoechst 33258 (C, blue fluorescence). Panel D is a merged image. Scale bar = 200 μm. (TIFF 559 KB) [file 12868_2014_3787_MOESM1_ESM.tiff]
